# Supplementary material for: Enabling long-cycling aqueous sodium-ion batteries via Mn dissolution inhibition using sodium ferrocyanide electrolyte additive
Source: Nat Commun. 2023 Jun 16;14:3591. doi: 10.1038/s41467-023-39385-6 (PMC10275921; doi:10.1038/s41467-023-39385-6)
Supplement: Supplementary file 1 — Supplementary Information [file 41467_2023_39385_MOESM1_ESM.pdf]

Supplementary Information

Enabling long-cycling aqueous sodium-ion batteries via Mn dissolution inhibition using sodium ferrocyanide electrolyte additive

Zhaoheng Liang, Fei Tian, Gongzheng Yang*, Chengxin Wang*

*Correspondence and requests for materials should be addressed to C. X. Wang and G. Z. Yang.

State Key Laboratory of Optoelectronic Materials and Technologies, School of Materials Science and Engineering, Sun Yat-sen (Zhongshan) University, Guangzhou 510275, PR China. e-mail: wchengx@mail.sysu.edu.cn; yanggzh5@mail.sysu.edu.cn

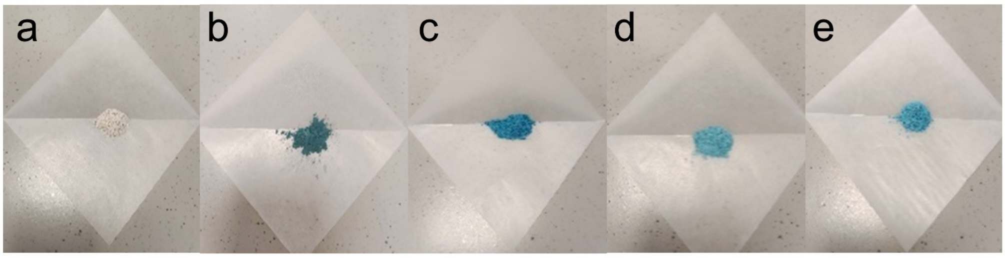

Supplementary Fig. 1 Optical images of PB-S1, PB-S2, PB-S3, PB-S4, PB-S5 from left to right. **a** The image of PB-S1. **b** The image of PB-S2. **c** The image of PB-S3. **d** The image of PB-S4. **e** The image of PB-S5. In this figure and the following figures, PB-S1 represents an as-prepared Mn-based Prussian blue. PB-S2 represents an modified Mn-based Prussian blue by HNO_3. PB-S3 represents an modified Mn-based Prussian blue by HNO_3 and $Na_4Fe(CN)_6$. PB-S4 represents an modified Mn-based Prussian blue by HNO_3 and Na_2SO_4. PB-S5 represents an modified Mn-based Prussian blue by HNO_3, $Na_4Fe(CN)_6$ and Na_2SO_4.

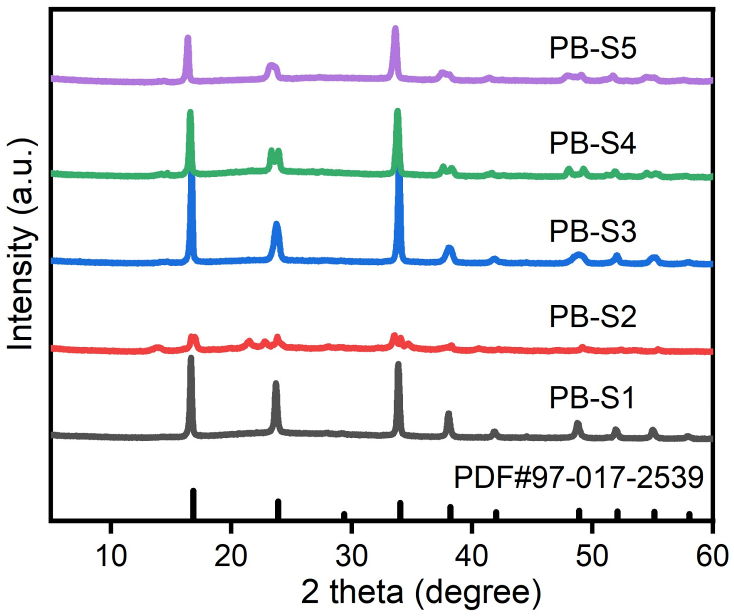

Supplementary Fig. 2 XRD patterns of different powder samples.

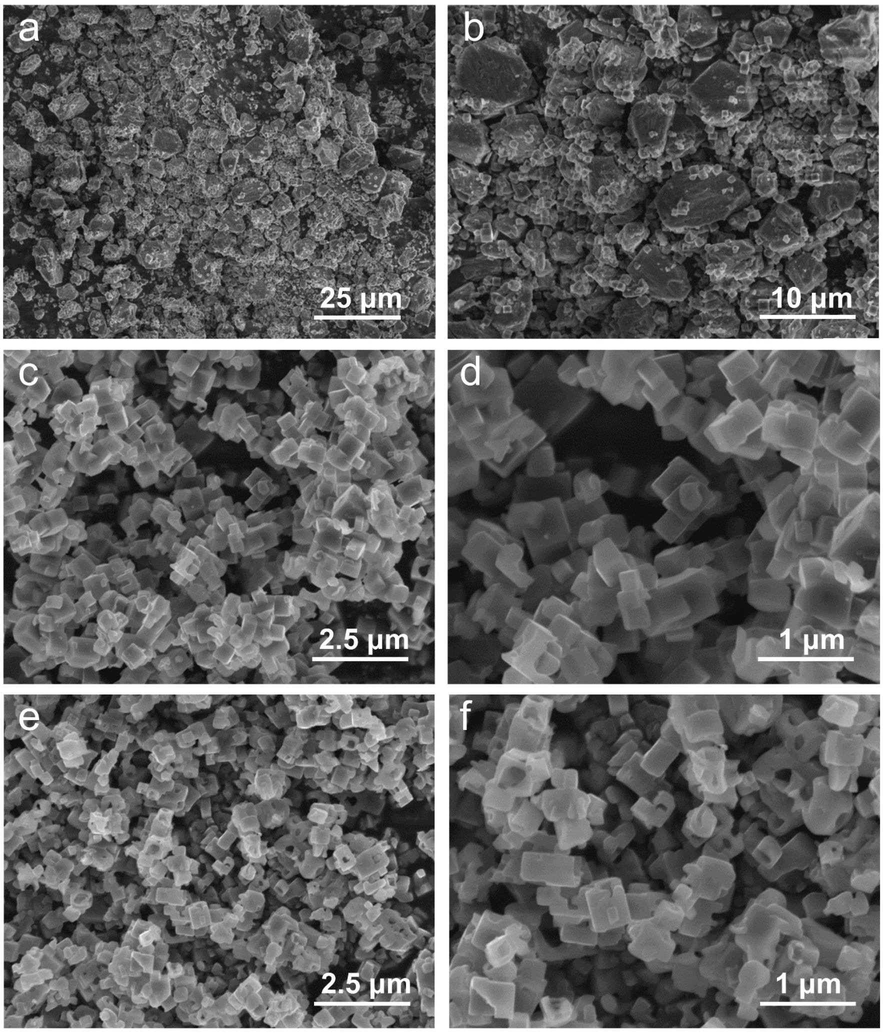

Supplementary Fig. 3 SEM images of different powder samples. **a-b** PB-S2. **c-d** PB-S3. **e-f** PB-S4.

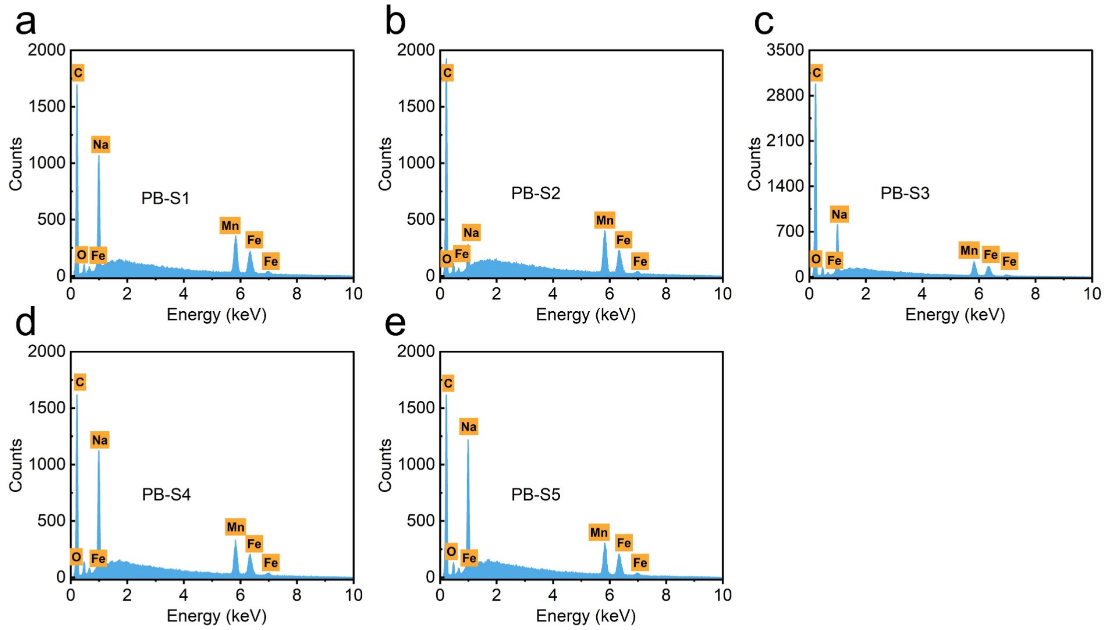

Supplementary Fig. 4 EDS results of different powder samples. **a** PB-S1 powder sample. **b** PB-S2 powder sample. **c** PB-S3 powder sample. **d** PB-S4 powder sample. **e** PB-S5 powder sample.

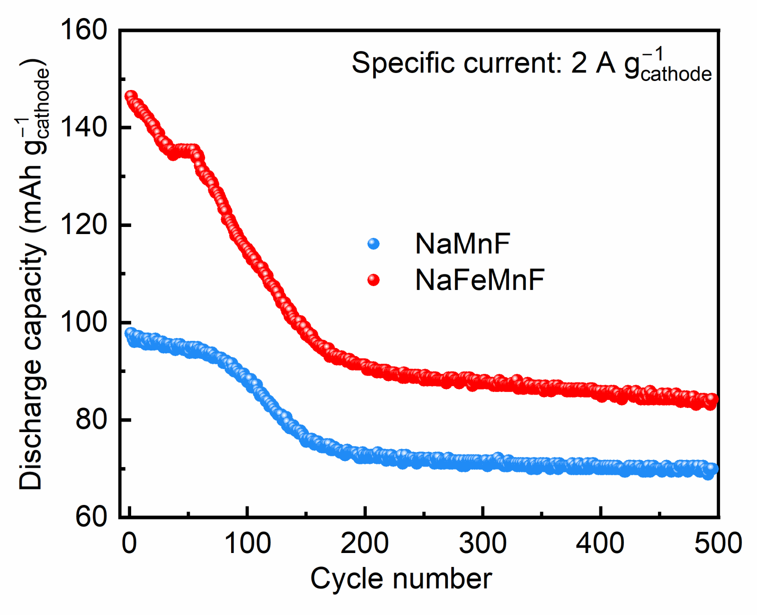

Supplementary Fig. 5 Cycling performance of PTCDI||NaMnF and PTCDI||NaFeMnF in the blank electrolyte at 25 °C.

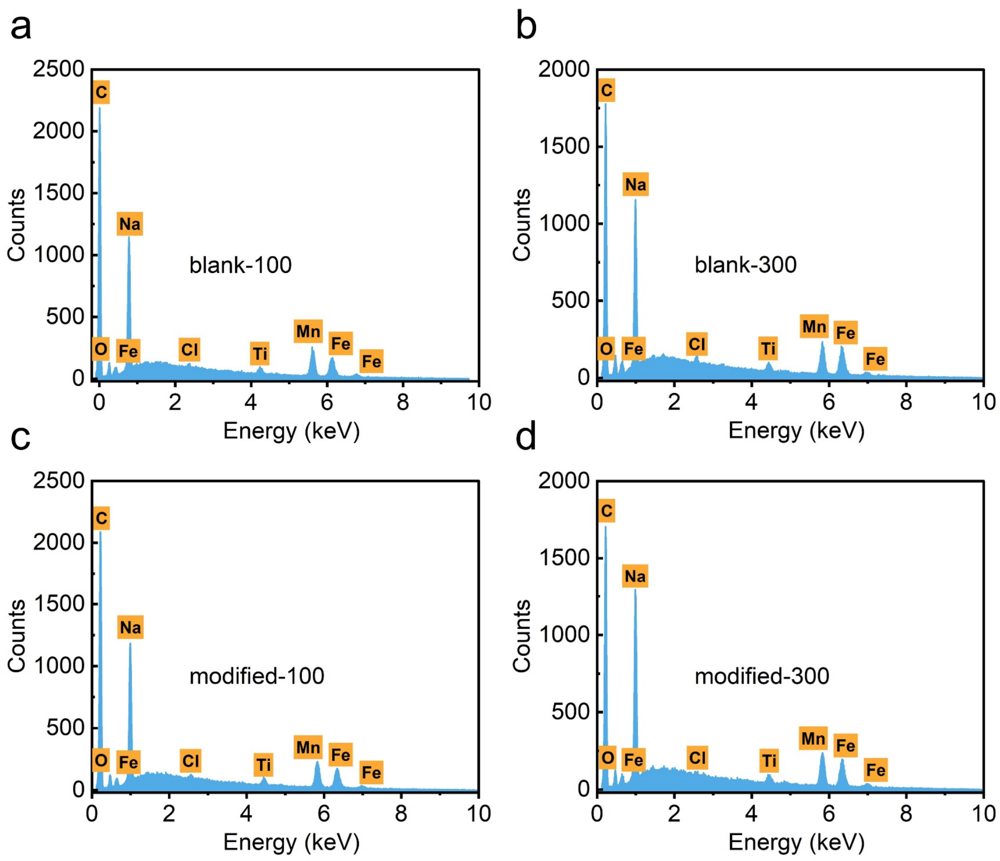

Supplementary Fig. 6 Ex situ EDS analysis of positive electrodes disassembled from PTCDI||NaFeMnF after certain cycles at 0.5 A g^{-1} at 25 °C in various electrolytes. **a** The electrode after 100 cycles in the blank electrolyte. **b** The electrode after 100 cycles in the modified electrolyte. **c** The electrode after 300 cycles in the blank electrolyte. **d** The electrode after 300 cycles in the modified electrolyte.

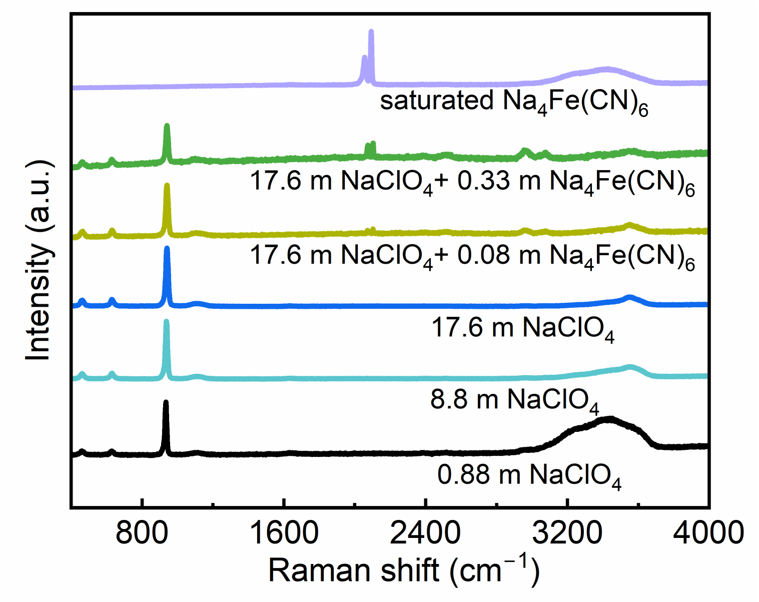

Supplementary Fig. 7 Raman spectra ranging from 400–4000 cm^{-1} of various electrolyte formulations.

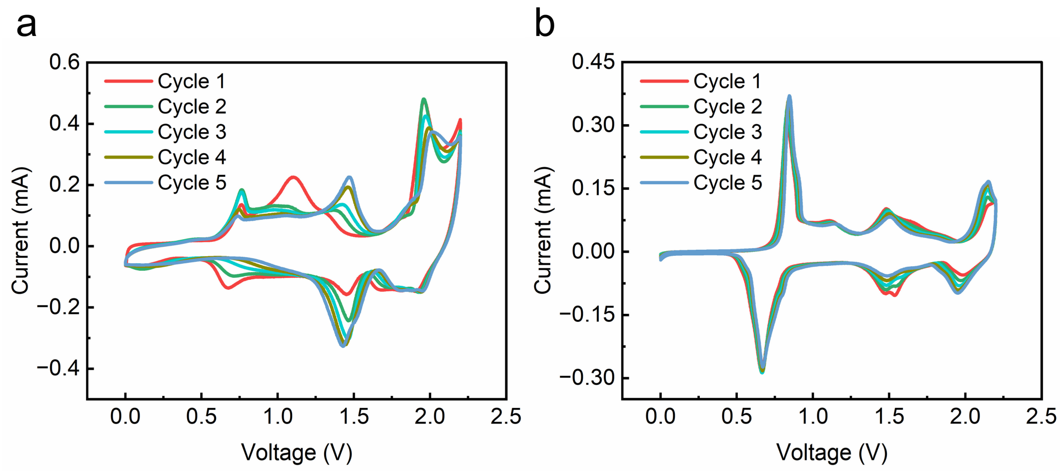

Supplementary Fig. 8 a CV curves of PTCDI||NaMnF in the blank electrolyte at 25 °C. **b** CV curves of PTCDI||NaFeMnF in the modified electrolyte at 25 °C.

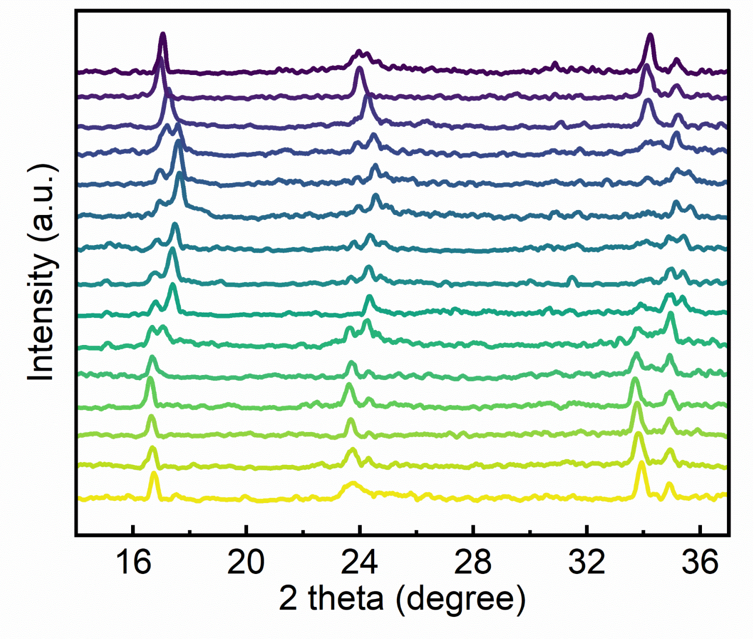

Supplementary Fig. 9 In situ XRD patterns of NaFeMnF in PTCDI||NaFeMnF in the modified electrolyte during charge-discharge processes at 25 °C.

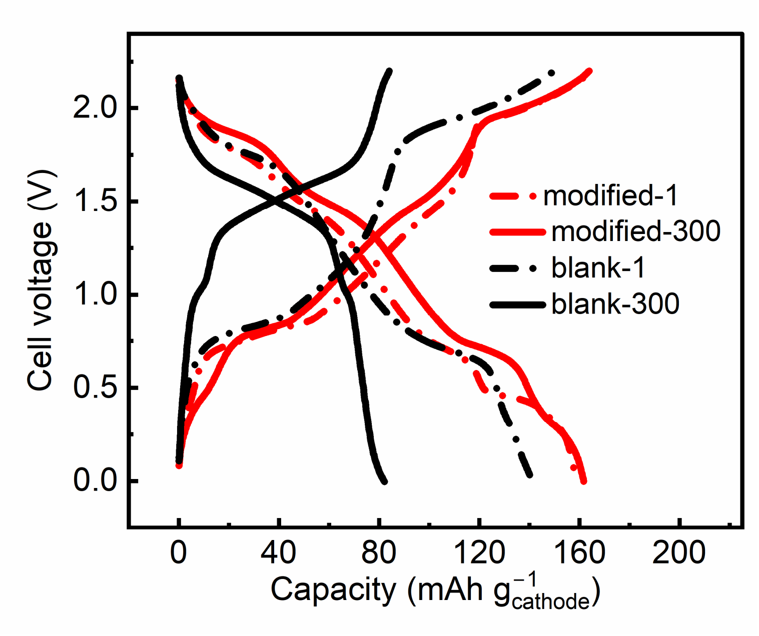

Supplementary Fig. 10 The 1st and 300th charge-discharge profiles of PTCDI||NaFeMnF in the blank electrolytes and modified electrolyte at 0.5 A g^{-1} at 25 °C.

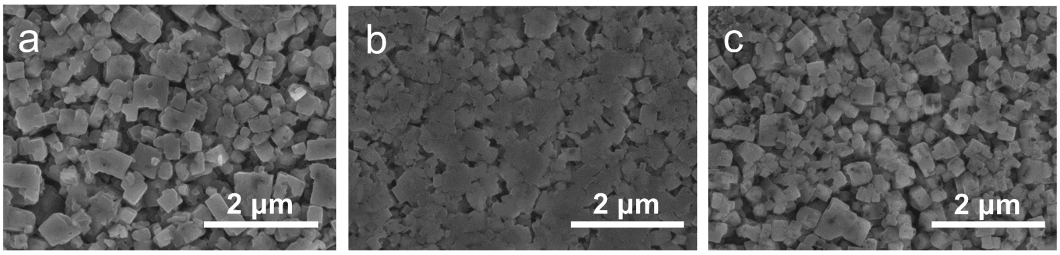

Supplementary Fig. 11 Ex situ SEM measurements of positive electrodes disassembled from PTCDI||NaFeMnF before and after cycling in various electrolytes at 0.5 A g^{-1} at 25 °C. **a** The pristine electrode. **b** The electrode after 100 cycles in the blank electrolyte. **c** The electrode after 100 cycles in the modified electrolyte.

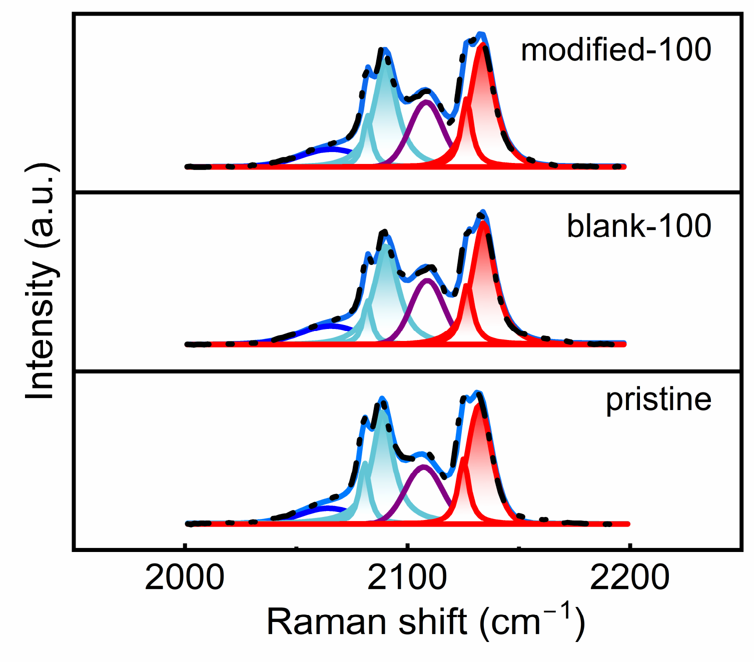

Supplementary Fig. 12 Ex situ Raman measurements of fully discharged positive electrodes disassembled from PTCDI||NaFeMnF before and after 100 cycles in blank and modified electrolytes at 0.5 A g^{-1} at 25 °C. The blue peaks correspond to Fe^{2+}–C≡N–Mn^{2+} vibration mode and the red peaks correspond to Fe^{2+}–C≡N–Mn^{3+} vibration mode.

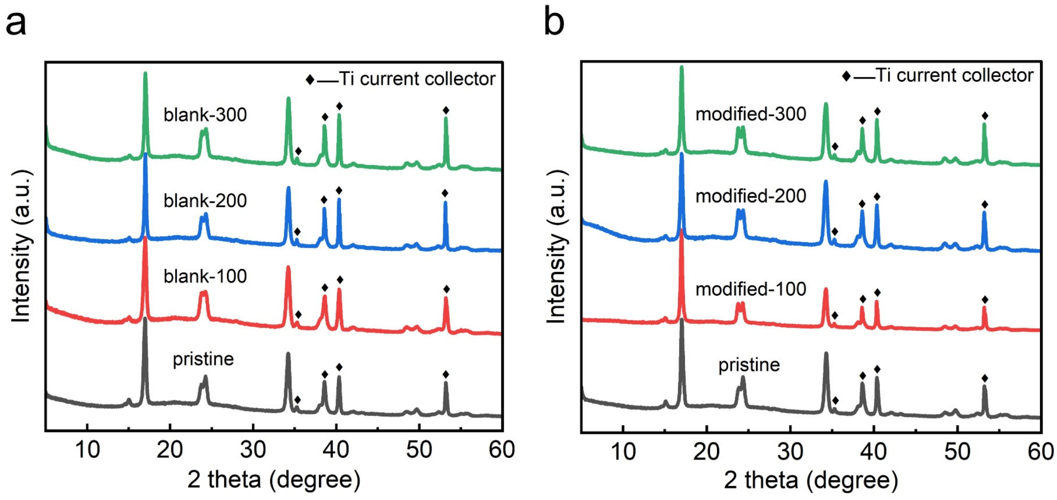

Supplementary Fig. 13 Ex situ XRD measurements of fully discharged positive electrodes disassembled from PTCDI||NaFeMnF before and after cycling in various electrolytes at 0.5 A g^{-1} at 25 °C. **a** The comparison of the electrode after different cycles in the blank electrolyte. **b** The comparison of the electrode after different cycles in the modified electrolyte.

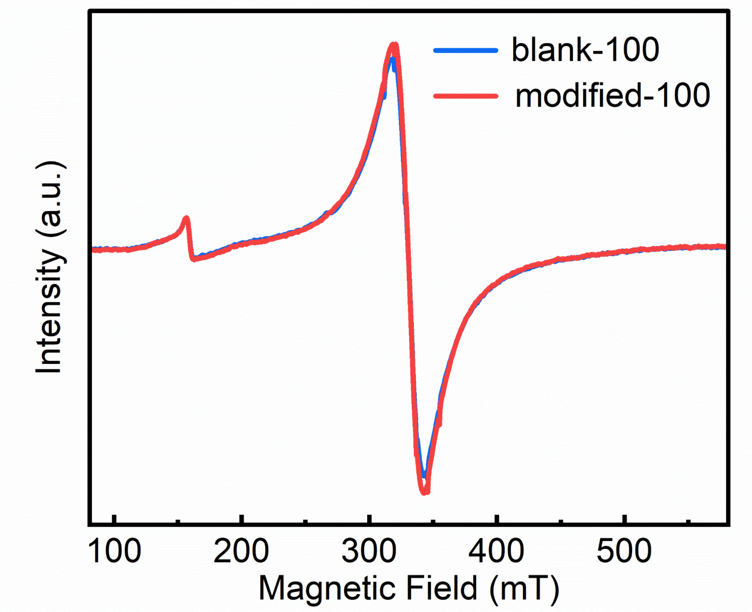

Supplementary Fig. 14 Ex situ EPR measurements of fully discharged positive electrodes disassembled from PTCDI||NaFeMnF after 100 cycles in the blank and modified electrolytes at 0.5 A g^{-1} at 25 °C.

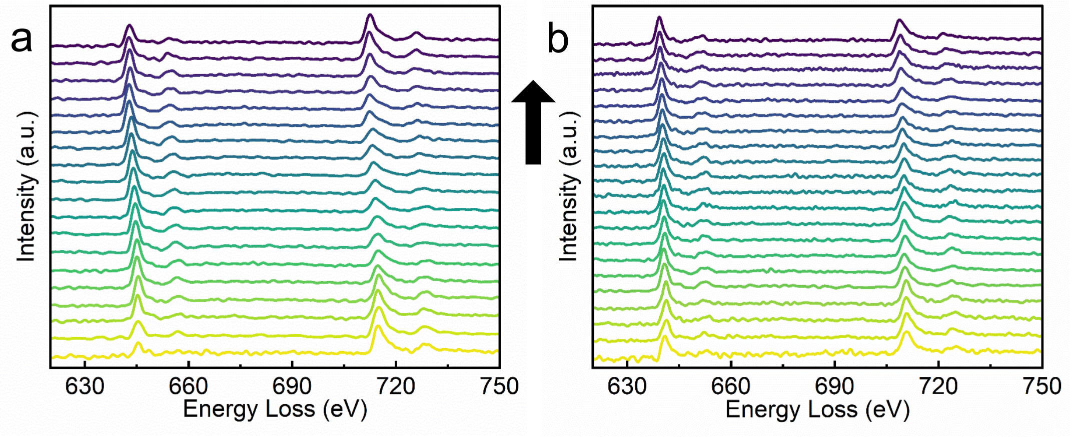

Supplementary Fig. 15 Ex situ EELS measurements of fully discharged positive electrodes disassembled from PTCDI||NaFeMnF after 300 cycles in the various electrolytes at 0.5 A g^{-1} at 25 °C. **a** The cycled electrode in the blank electrolyte. **b** The cycled electrode in the modified electrolyte. The black arrows show the direction of scanning, which is from the exterior to interior and finally to the exterior.

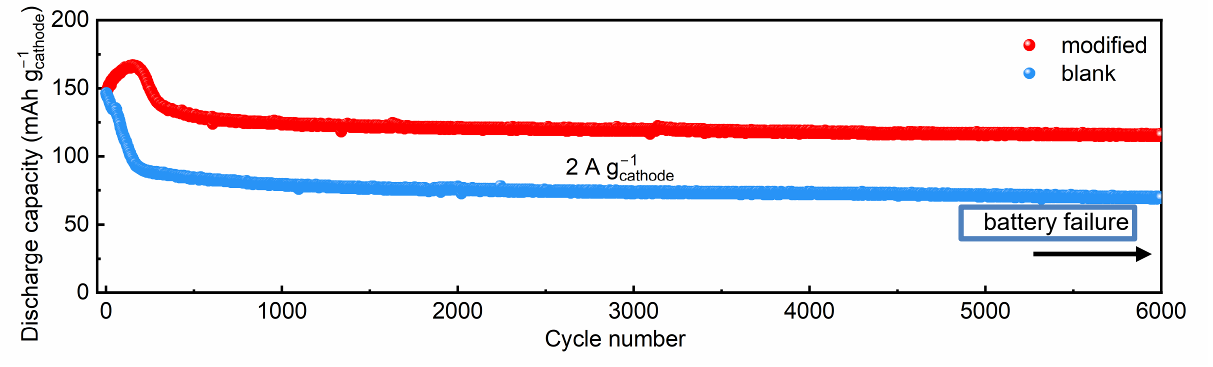

Supplementary Fig. 16 Cycling performance of PTCDI||NaFeMnF in the blank electrolyte and the modified electrolyte at 2 A g^{-1} at 25 °C.

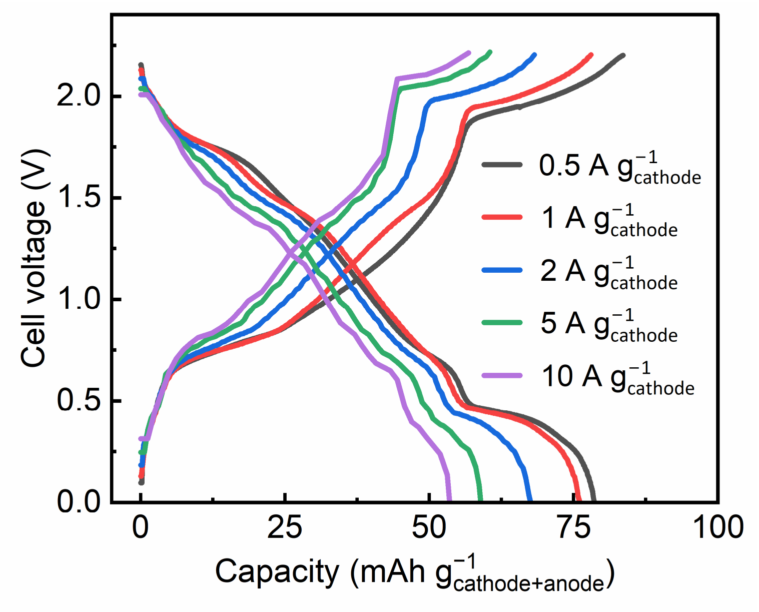

Supplementary Fig. 17 Charge-discharge curves of PTCDI||NaFeMnF in the modified electrolyte at different specific currents from 0.5 to 10 A g^{-1} at 25 °C.

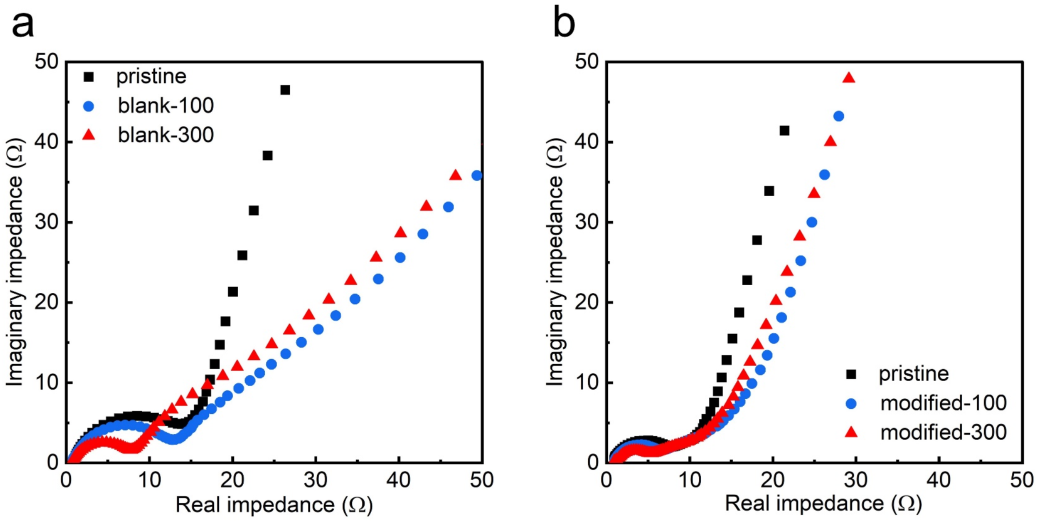

Supplementary Fig. 18 The EIS (only qualitative evaluation) of fully discharged positive electrodes disassembled from PTCDI||NaFeMnF after cetain cycles in the various electrolytes at 0.5 A g^{-1} at 25 °C. **a** The comparison of the electrode after different cycles in the blank electrolyte. **b** The comparison of the electrode after different cycles in the modified electrolyte.

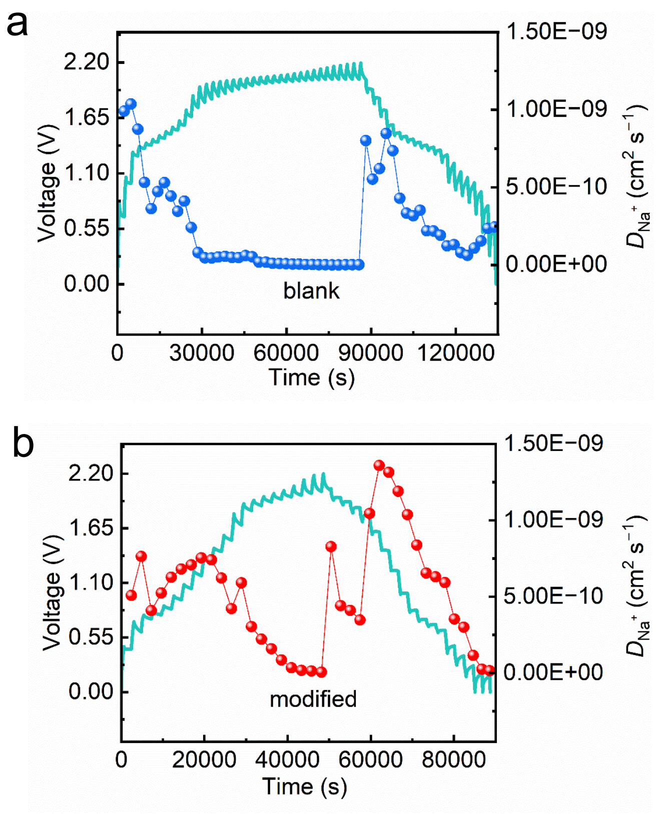

Supplementary Fig. 19 The GITT results of PTCDI||NaFeMnF cells in various electrolytes. **a** PTCDI||NaFeMnF in the blank electrolyte. **b** PTCDI||NaFeMnF in the modified electrolyte. The measurements were conducted during the first cycle after activation at specific current of 40 mA g^{-1}, in which the cell was alternately charged for 10 min followed by 40 min resting.

Supplementary Table 1 Mass loss of samples after different modifications. In the following tables, PB-S1 represents an as-prepared Mn-based Prussian blue. PB-S2 represents an modified Mn-based Prussian blue by HNO_3. PB-S3 represents an modified Mn-based Prussian blue by HNO_3 and $Na_4Fe(CN)_6$. PB-S4 represents an modified Mn-based Prussian blue by HNO_3 and Na_2SO_4. PB-S5 represents an modified Mn-based Prussian blue by HNO_3, $Na_4Fe(CN)_6$ and Na_2SO_4. The m_1 represents the mass of the reactant before modification. The m_2 represents the mass of the products after modification. The mass loss represents $m_2/m_1*100\%$, reflecting the mass loss after different modifications.

Before modification		additives	After modification		
Reactant	Mass (m_1)		Product	Mass (m_2)	Mass loss
PB-S1	1 g	HNO_3	PB-S2	0.475 g	52.5%
		HNO_3 + $Na_4Fe(CN)_6$	PB-S3	0.85 g	15%
		HNO_3 + Na_2SO_4	PB-S4	0.7 g	30%
		HNO_3 + $Na_4Fe(CN)_6$ + Na_2SO_4	PB-S5	1 g	0%

Supplementary Table 2 The atomic ratio of different samples obtained from EDS analysis. The measurements errors indicate a 68.4% confidence interval.

Sample	Na	Mn	Fe	Mn:Fe	Na:(Mn + Fe)
PB-S1	13.77 ± 0.56	17.50 ± 1.14	16.18 ± 1.19	1.08	0.41
PB-S2	2.73 ± 0.13	20.95 ± 1.24	17.73 ± 1.19	1.18	0.07
PB-S3	7.14 ± 0.37	9.83 ± 0.81	11.45 ± 1.06	0.86	0.34
PB-S4	14.15 ± 0.57	16.09 ± 1.05	15.95 ± 1.17	1.01	0.44
PB-S5	15.10 ± 0.61	14.98 ± 0.99	16.42 ± 1.22	0.92	0.48

Supplementary Table 3 Refined parameters for NaMnF.

S. G. Fm-3m, a=b=c=10.5123 Å, α=β=γ=91.667°					
Atoms	x	y	z	B	Occ.
C1	0.00000(0)	0.00000(0)	0.17630(0)	0.921(0)	0.96
Mn1	0.00000(0)	0.00000(0)	0.50000(0)	0.621(0)	1.00
Fe1	0.00000(0)	0.00000(0)	0.00000(0)	1.299(0)	0.96
Na1	0.25000(0)	0.25000(0)	0.25000(0)	3.613(0)	0.665
N1	0.00000(0)	0.00000(0)	0.29396(0)	3.987(0)	0.160
O1	0.00000(0)	0.00000(0)	0.27130(0)	6.535(0)	1.000
O2	0.18972(0)	0.18972	0.18972(0)	5.965(0)	0.068
O3	0.25000(0)	0.25000(0)	0.25000(0)	5.067(0)	0.335
O4	0.24994(0)	-0.05939(0)	0.24361(0)	0.554(0)	0.596

Supplementary Table 4 Refined parameters for NaFeMnF.

S. G. P21/n, a=10.51710 Å, b=7.44888 Å, c=7.42714 Å, β=91.81326°					
Atoms	x	y	z	B	Occ.
Mn1	0.50000(0)	0.50000(0)	0.50000(0)	0.41400(0)	0.93000
Fe1	0.50000(0)	0.50000(0)	0.50000(0)	0.84000(0)	0.07000
Fe2	0.50000(0)	0.00000(0)	1.00000(0)	0.84000(0)	0.96000
Na1	0.22387(0)	0.46902	-0.01918(0)	3.00000(0)	0.76000
Na2	0.49386(0)	0.27671(0)	0.70509(0)	3.00000(0)	0.76000
O1	0.23872(0)	0.04901(0)	0.54193(0)	0.56400(0)	0.98000
O2	0.77510(0)	1.02030(0)	1.00829(0)	0.56400(0)	0.98000
O3	0.00601(0)	0.28873(0)	0.71241(0)	0.56400(0)	0.98000
O4	0.25069(0)	0.78498(0)	0.28904(0)	0.56400(0)	0.98000
O5	1.26284(0)	1.12596(0)	0.37439(0)	0.56400(0)	0.98000
O6	0.97864(0)	1.00164(0)	0.54357(0)	0.56400(0)	0.98000
C1	0.49997(0)	0.19817(0)	0.79630(0)	0.55300(0)	0.96000
N1	0.51109(0)	0.27144(0)	0.72479(0)	0.72600(0)	0.96000
C2	0.21805(0)	0.51114(0)	0.52613(0)	0.55300(0)	0.96000
N2	0.27553(0)	0.49293(0)	0.50933(0)	0.72600(0)	0.96000
C3	0.50205(0)	0.19575(0)	0.21147(0)	0.55300(0)	0.96000
N3	0.51456(0)	0.26448(0)	0.24582(0)	0.72600(0)	0.96000

Supplementary Table 5 The bulk atomic ratio obtained from ICP-AES.

Sample	Weight %			Relative atomic ratio		
	Na	Mn	Fe	Na	Mn	Fe
NaMnF	21.91	39.58	38.51	1.39	1.04	1
NaFeMnF	24.32	35.68	40.01	1.58	0.97	1.07

Supplementary Table 6 The atomic ratio of different electrodes obtained from EDS analysis. The measurements errors indicate the 68.4% confidence interval.

Sample	Na	Mn	Fe	Mn:Fe	Na:(Mn + Fe)
blank-100	11.61 ± 0.54	10.38 ± 0.85	13.13 ± 1.07	0.79	0.49
modified-100	12.30 ± 0.56	11.02 ± 0.83	12.71 ± 1.07	0.87	0.52
blank-300	13.31 ± 0.53	10.59 ± 0.70	15.86 ± 1.14	0.67	0.50
modified-300	15.23 ± 0.64	12.36 ± 0.86	14.30 ± 1.11	0.86	0.57

Supplementary Table 7 The comparison of some ASIBs previously reported with this work. In the below table, the m represents molality (mol kg^{-1}). The M represents molarity (mol L^{-1}). The specific energy was calculated based on the active materials mass of both electrodes.

Cell configuration	Temperature (°C)	Electrolyte	Coulombic efficiency (%)	Discharge capacity (mAh g^{-1})	Average discharge voltage (V)	Specific energy (Wh kg^{-1})	Cycling stability	Reference
$Na_3MnTi(PO_4)_3$‖ $Na_3MnTi(PO_4)_3$	room temperature	1 M Na_2SO_4 in H_2O	99	60	1.4	40	98%, 100 cycles	1
NTP‖ $Na_4Fe_3(PO_4)_2(P_2O_7)$	25	17 m $NaClO_4$ in H_2O	99	85	1	36	75%, 200 cycles	2
NTP@C‖ $Na_{0.44}MnO_2$	room temperature	$NaClO_4 \cdot H_2O$, H_2O and urea molar ratio of 1:3:2	100	125	1	50	90.0%, 3500 cycles	3
NTP‖ $Na_{1.88}Mn[Fe(CN)_6]_{0.97} \cdot 1.35H_2O$	–	9 m NaOTF + 22 m TEAOTF	97	140	1.74	71	76%, 800 cycles	4
PTCDI‖NaFeMnF	25	17 m $NaClO_4$ in H_2O	100	155	1.35	94	73.4%, 15000 cycles	This work

Supplementary Table 8 The comparison of other ASIBs previously reported with this work. In the below table, The N/P represents mass ratio of the negative electrode and the positive electrode. The m represents molality (mol kg^{-1}). The M represents molarity (mol L^{-1}). The specific energy was calculated based on the active materials mass of both electrodes.

Cell configuration	Temperature (°C)	Mass loading (mg cm^{-2}), N/P ratio	Electrolyte	Capacity (mAh g^{-1})	Average discharge voltage (V)	Specific energy (Wh kg^{-1})	Cycling stability	Reference
NTP|| $Na_4Fe_3(PO_4)_2(P_2O_7)$	25	–, 1:1	17 m $NaClO_4$ in H_2O	85	1	36	75%, 200 cycles	2
NTP@C|| $Na_{0.44}MnO_2$	room temperature	7.5, 1.65:1	$NaClO_4$·H_2O , H_2O and urea molar ratio of 1:3:2	75	1	50	90%, 3500 cycles	3
NTP|| $Na_{1.88}Mn[Fe(CN)_6]_{0.97}$·$1.35H_2O$	–	–, 1.97:1	9 m NaOTF + 22 m TEAOTF	140	1.74	71	76%, 800 cycles	4
NTP||NaCoPB	room temperature	7, 1.3:1	1 M Na_2SO_4 in H_2O	128	1.33	67	98%, 100 cycles	5
NTP||NaCuPB	–	7, 1:1.7	1 M Na_2SO_4 in H_2O	57	1.4	48	88%, 1000 cycles	6
KHMCC||NaMnPB	–	–, 2:1	17 m $NaClO_4$ in H_2O	110	1.7	58	78%, 100 cycles	7
NTP||NaMnPB	room temperature	–, 1:1	2.5 SN/ $NaClO_4$·H_2O molar ratio of 2.5:1	135	1.9	80	74.5%, 1000 cycles	8
PTCDI||NaFeMnF	25	1.0–2.0, 1.25:1	17 m $NaClO_4$ in H_2O	157	1.35	94	73.4%, 15000 cycles	this work

Supplementary Note 1

Comparison of different modifications and the design of the electrolyte

All the samples were characterized by SEM (Supplementary Fig. 3). By controlling experimental conditions during synthesis process, it can be found that Na^+ serves as protectant and $Fe(CN)^{4-}$ plays the role of an iron-doping dopant for PB-S1 during the etching process. As seen in Supplementary Fig. 3a, b, PB-S2 showed an irregular morphology; in contrast, PB-S3 exhibited a cube-like morphology (Supplementary Fig. 3c, d). This demonstrates that $Na_4Fe(CN)_6$ is able to prevent particles from etching. In addition, PB-S3 particles show more square edges and corners than PB-S1 particles. To identify the effects of Na^+, Na_2SO_4 was added alone during the etching process, from which PB-S4 was obtained. As seen in Supplementary Fig. 3e, f, PB-S4 is composed of nanometre-scale cubes with a porous structure on the surface but still maintains the structure of the pristine material. The result implies that the addition of Na ions is indeed helpful to protect the structure from nitric acid. To gain insight into the composition of different samples, EDS was carried out, as shown in Supplementary Fig. 4 and Supplementary Table 2. The ratio of Na to (Mn + Fe) for PB-S4 is 0.44, significantly higher than that of PB-S2, indicating that additional Na ions could inhibit oxidation by nitric acid. This can also be verified by the mass loss after various modifications, as shown in Supplementary Table 1. In addition, the lower ratio of Mn to Fe for PB-S3 suggests that Fe-doped NaMnPB was probably obtained in the presence of $Na_4Fe(CN)_6$. A detailed discussion is presented in the manuscript. Notably, when $Na_4Fe(CN)_6$ and Na_2SO_4 were both added (synthesis route for PB-S5), mass loss by etching was minimized, as Supplementary Table 1 shows. From this perspective, we added $Na_4Fe(CN)_6$ to the highly concentrated $NaClO_4$ solution as a modified electrolyte to offer maximum protection for the cathode.

Supplementary Note 2

Comparison of PTCDI||NaMnF and PTCDI||NaFeMnF in the blank electrolyte

To investigate electrochemical behaviour, cyclic voltammetry measurements were performed for PTCDI||NaMnF and PTCDI||NaFeMnF in the blank solution. The two curves have nearly the same peak positions. In this case, it is noteworthy that the redox pair at approximately 1.5 V is derived from Mn^{2+}/Mn^{3+} with split energy levels instead of from Fe^{2+}/Fe^{3+} coordinated with C or N. In addition, during the repeated scans, the CVs of PTCDI||NaMnF change gradually, while the CVs of PTCDI||NaFeMnF nearly overlap, indicating different cycling performances.

Supplementary Note 3

Calculation of the Na^+ diffusion coefficient

GITT measurements were performed to analyse the kinetic properties of Na^+. The measurements were conducted during the first cycle after activation at a current density of 40 mA g^{-1}, in which the cell was alternately charged for 10 min followed by 40 min of rest. The Na^+ diffusion coefficient can be calculated by the following equation:

$$D_{Na^+}=\frac{4}{\pi\tau}\left(\frac{m_B V_m}{M_B S}\right)^2\left(\frac{\Delta E_S}{\Delta E_\tau}\right)^2 \tag{1}$$

where τ is the duration of the current pulse, m_B is the mass of the active material, V_m is the molar volume of the electrode, M_B is the molar mass of the material, S is the contact area between the electrode and electrolyte, ΔE_s is the steady-state voltage change, and ΔE_τ is the voltage change during the constant current pulse.

Supplementary References

1. Gao, H. & Goodenough, J. B. An Aqueous Symmetric Sodium-Ion Battery with NASICON-Structured $Na_3MnTi(PO_4)_3$. *Angew. Chem. Int. Ed.* **128**, 12960–12964 (2016).
2. Lee, M. H. *et al.* Toward a low-cost high-voltage sodium aqueous rechargeable battery. *Mater. Today* **29**, 26–36 (2019).
3. Hou, Z. *et al.* Towards High-Performance Aqueous Sodium Ion Batteries: Constructing Hollow $NaTi_2(PO_4)_3$@C Nanocube Anode with Zn Metal-Induced Pre-Sodiation and Deep Eutectic Electrolyte. *Adv. Energy Mater.* **12**, 2104053 (2022).
4. Jiang, L. *et al.* High-Voltage Aqueous Na-Ion Battery Enabled by Inert-Cation-Assisted Water-in-Salt Electrolyte. *Adv. Mater.* **32**, 1904427 (2020).
5. Wu, X. *et al.* Vacancy-Free Prussian Blue Nanocrystals with High Capacity and Superior Cyclability for Aqueous Sodium-Ion Batteries. *ChemNanoMat* **1**, 188–193 (2015).
6. Wu, X. Y. *et al.* Energetic aqueous rechargeable sodium-ion battery based on $Na_2CuFe(CN)_6$-$NaTi_2(PO_4)_3$ intercalation chemistry. *ChemSusChem* **7**, 407–411 (2014).
7. Nakamoto, K., Sakamoto, R., Sawada, Y., Ito, M. & Okada, S. Over 2 V Aqueous Sodium-Ion Battery with Prussian Blue-Type Electrodes. *Small Methods* **3**, 1800220 (2019).
8. Liu, T. *et al.* Water-Locked Eutectic Electrolyte Enables Long-Cycling Aqueous Sodium-Ion Batteries. *ACS Appl. Mater. Interfaces* **14**, 33041–33051 (2022).
