# Supplementary material for: Enabling long-cycling aqueous sodium-ion batteries via Mn dissolution inhibition using sodium ferrocyanide electrolyte additive
Source: Nat Commun. 2023 Jun 16;14:3591. doi: 10.1038/s41467-023-39385-6 (PMC10275921; doi:10.1038/s41467-023-39385-6)
Supplement: Supplementary file 3 — Reporting Summary [file 41467_2023_39385_MOESM3_ESM.pdf]

nature portfolio

Corresponding author(s): Gongzheng Yang, Chengxin Wang

Last updated by author(s): Jun 2, 2023

Reporting Summary

Nature Portfolio wishes to improve the reproducibility of the work that we publish. This form provides structure for consistency and transparency in reporting. For further information on Nature Portfolio policies, see our Editorial Policies and the Editorial Policy Checklist.

Statistics

For all statistical analyses, confirm that the following items are present in the figure legend, table legend, main text, or Methods section.

n/a	Confirmed	
☒	☐	The exact sample size (*n*) for each experimental group/condition, given as a discrete number and unit of measurement
☐	☒	A statement on whether measurements were taken from distinct samples or whether the same sample was measured repeatedly
☒	☐	The statistical test(s) used AND whether they are one- or two-sided<br>*Only common tests should be described solely by name; describe more complex techniques in the Methods section.*
☒	☐	A description of all covariates tested
☐	☒	A description of any assumptions or corrections, such as tests of normality and adjustment for multiple comparisons
☐	☒	A full description of the statistical parameters including central tendency (e.g. means) or other basic estimates (e.g. regression coefficient) AND variation (e.g. standard deviation) or associated estimates of uncertainty (e.g. confidence intervals)
☒	☐	For null hypothesis testing, the test statistic (e.g. *F*, *t*, *r*) with confidence intervals, effect sizes, degrees of freedom and *P* value noted<br>*Give P values as exact values whenever suitable.*
☒	☐	For Bayesian analysis, information on the choice of priors and Markov chain Monte Carlo settings
☒	☐	For hierarchical and complex designs, identification of the appropriate level for tests and full reporting of outcomes
☒	☐	Estimates of effect sizes (e.g. Cohen's *d*, Pearson's *r*), indicating how they were calculated

Our web collection on statistics for biologists contains articles on many of the points above.

Software and code

Policy information about availability of computer code

Data collection	VESTA VERSION 3.1.9 was used to generate and edit crystal cell structure.
Data analysis	See above.

For manuscripts utilizing custom algorithms or software that are central to the research but not yet described in published literature, software must be made available to editors and reviewers. We strongly encourage code deposition in a community repository (e.g. GitHub). See the Nature Portfolio guidelines for submitting code & software for further information.

Data

Policy information about availability of data

All manuscripts must include a data availability statement. This statement should provide the following information, where applicable:

- Accession codes, unique identifiers, or web links for publicly available datasets
- A description of any restrictions on data availability
- For clinical datasets or third party data, please ensure that the statement adheres to our policy

All the data generated in this study are provided in the Source Data file. Source data are provided with this paper.

Human research participants

Policy information about studies involving human research participants and Sex and Gender in Research.

Reporting on sex and gender	Sex and gender were not involved or considered in this study.
Population characteristics	Covariate-relevant population characteristics of the human research participants were not involved in this study.
Recruitment	No participants were recruited in this study.
Ethics oversight	No relevant organizations were involved.

Note that full information on the approval of the study protocol must also be provided in the manuscript.

Field-specific reporting

Please select the one below that is the best fit for your research. If you are not sure, read the appropriate sections before making your selection.

☐ Life sciences ☐ Behavioural & social sciences ☒ Ecological, evolutionary & environmental sciences

For a reference copy of the document with all sections, see nature.com/documents/nr-reporting-summary-flat.pdf

Ecological, evolutionary & environmental sciences study design

All studies must disclose on these points even when the disclosure is negative.

Study description	Fe-substituted Mn based Prussian blue was prepared and applied to aqueous sodium ion batteries for the first time. Sodium ferrocyanide was added into the electrolyte as a supporting agent to alleviate the Mn dissolution. This work presented a battery performance of specific energy up to 94 Wh kg–1 (based on the active materials mass of both electrode) and 73.4% capacity retention after 15000 cycles.
Research sample	1. The prepared Prussian blue analogues (cathode materials) and electrolytes are the research samples.<br>2. Pristine electrodes and electrodes cycling in different electrolytes after a certain number of cycles are the samples.<br>3. For characterization, areas in micron scale on the electrode are the samples as well.<br>These were chosen to investigate the modification for materials, the changes of electrodes before and after cycling, and the electrolyte additive effect on cycled electrodes after certain number of cycles.
Sampling strategy	No specific sample-size calculation was performed. The samples were prepared for electrochemical tests or characterization on scanning electron microscopy, etc. Conclusions were drawn when the experimental results can be reproducible or follow a certain rule. Therefore, no specific sample-size calculation was performed.
Data collection	All electrochemical data are collected by Neware battery system and stored in real time. All characterization data were collected under appropriate test conditions.
Timing and spatial scale	The data were collected from February 28, 2022 to September 25, 2022 in Guangzhou, China.
Data exclusions	No data were excluded from the analysis.
Reproducibility	Ensure that no impurities are introduced during battery assembly and all characterizations are carried out under the full discharge state of the battery.
Randomization	1. The prepared Prussian blue analogues were grouped based on whether there was modification for the material. The electrolytes were grouped based on the salt and concentration.<br>2. Electrodes were grouped according to which electrolyte (the blank electrolyte with no additive or the modified electrolyte) they cycled in. For the cycled electrodes, grouping was based on the number of cycles.<br>3. For micron areas on the electrode, no grouping.
Blinding	Blinding was not involved in the study. Samples do not meet the condition for blinding.

Did the study involve field work? ☐ Yes ☒ No

Reporting for specific materials, systems and methods

We require information from authors about some types of materials, experimental systems and methods used in many studies. Here, indicate whether each material, system or method listed is relevant to your study. If you are not sure if a list item applies to your research, read the appropriate section before selecting a response.

Materials & experimental systems

n/a	Involved in the study
☒	☐ Antibodies
☒	☐ Eukaryotic cell lines
☒	☐ Palaeontology and archaeology
☒	☐ Animals and other organisms
☒	☐ Clinical data
☒	☐ Dual use research of concern

Methods

n/a	Involved in the study
☒	☐ ChIP-seq
☒	☐ Flow cytometry
☒	☐ MRI-based neuroimaging
